# Childhood otitis media: Relationship with daycare attendance, harsh parenting, and maternal mental health

**DOI:** 10.1371/journal.pone.0219684

**Published:** 2019-07-16

**Authors:** Kai-Wei Kevin Chen, Daniel Tsung-Ning Huang, Li-Tuan Chou, Hsi-Ping Nieh, Ren-Huei Fu, Chien-Ju Chang

**Affiliations:** 1 Division of Infectious Diseases, Department of Pediatrics, MacKay Children's Hospital, Taipei City, Taiwan, R.O.C; 2 Department of Medicine, MacKay Medical College, Sanzhi District, New Taipei City, Taiwan, R.O.C; 3 Department of Human Development and Family Studies, National Taiwan Normal University, Taipei City, Taiwan, R.O.C; 4 Chang Gung Medical Education Research Center, Division of Neonatology, Department of Pediatric, Chang Gung Memorial Hospital Linkou Branch, Guishan District, Taoyuan City, Taiwan, R.O.C; Harvard TH Chan School of Public Health, UNITED STATES

## Abstract

Psychological stress has been linked to developmental problems and poor health in children, but it is unclear whether it is also related to otitis media (OM). As part of a long-term study surveying the characteristics of childcare and development in Taiwan, we analyzed the relationship between OM and sources of psychological stress in children, such as poor maternal mental health and harsh parental discipline. We analyzed the data of 1998 children from the “Kids in Taiwan: National Longitudinal Study of Child Development & Care (KIT) Project” at the age of 3 years. Using bivariate and multivariate logistic regression models, we tested several risk factors as potential independent predictors of two outcomes: parent-reported incidence of OM and child health. The proportion of children who had developed OM in the first 3 years of their life was 12.5%. Daycare attendance (odds ratio [OR]: 1.475; 95% confidence interval [CI]: 1.063–2.046), poor maternal mental health (OR: 1.913; 95% CI: 1.315–2.784), and harsh parental discipline (OR: 1.091; 95% CI: 1.025–1.161) correlated with parent-reported occurrence of OM. These findings suggest that providing psychosocial support to both parents and children might be a novel strategy for preventing OM.

## Introduction

Otitis media (OM), which is defined as “an inflammation of the middle ear without reference to etiology or pathogenesis” [1], is one of the most common childhood infectious diseases. It is one of the leading reasons for clinic visits, antibiotic consumption, and surgical treatment in children [2]. The incidence of OM is substantial: 50–85% of children in the United States would have experienced acute OM by the age of 3 years [3–6]. Only few studies have investigated the epidemiology on OM in Taiwan. Although some of the studies showed the prevalence and incidence of OM among Taiwanese children to be lower than in other countries [7, 8], untreated OM may lead to severe complications such as sepsis, meningitis, or brain abscess, and recurrent OM may cause long-term sequelae such as hearing impairment and delayed speech development [2, 9]. Consequently, OM is a childhood disease with a significant health and economic burden, and identifying its predisposing factors can lead to early intervention and effective prevention of the disease.

The risk factors associated with OM are related to two components: the host’s immune response and the microbial load in the environment. Impaired immune system due to age, genetic disposition, or atopy are examples of host-related factors, while environmental factors, such as daycare attendance, are related to the microbial load [10]. Some of the most established risk factors include premature birth [11], short duration of breastfeeding [12–14], exposure to tobacco smoke [15], daycare attendance [16–18], and social disadvantages [19]. However, the relationship between OM and child psychological stress has been discussed in less depth. It is well known that psychological stress has a negative impact on health [20]; a stressful life has long been known to be a risk factor for several diseases, including immune and cardiovascular disorders [21–23]. Children experience different stresses at various stages of life. For children under the age of 3 years, the environment they live in and the people they interact with would be the main sources of psychological stress. Some examples of potential sources of psychological stress for children are poor parental mental health [24, 25], harsh parenting [26, 27], family poverty [28], and daycare attendance [29–32]. Interaction with parents could be a source of psychological stress for children; parental stress and harsh discipline might predispose children to increased psychological stress. There is accumulating evidence that parental exposure to stress is predictive of poor parent-reported child health [33, 34]. However, the relationship between OM and parental stress is not well studied. Parenting style could also have adverse effects on the development of children. Harsh discipline is linked with negative emotional and behavioral development in children [35–37]. Most previous reports have assessed the effects of harsh discipline on general health or psychosocial outcomes in children [26, 36, 38–40], and only few studies have focused on their effects on common childhood diseases. One study reported that childhood asthma morbidity might be higher when the mothers have poor mental health [41]. However, whether parental stress is also linked to OM has not been studied as intensively.

Taiwanese children experience different sources of stress. Taiwan has one of the lowest fertility rates in the world [42], and due to cultural reasons, Taiwanese parents tend to put extra attention and demands on their only child. This behavior imposes stress on both parents and children, but psychological stress has not been studied in depth as one of the possible host factors for the pathogenesis of OM. In the present study, we examined whether childhood psychological stress is related to the incidence of parent-reported OM. We also analyzed the relationship of psychological stress with parent-reported child health status as the secondary outcome. Data were drawn from a nationally representative sample of 1998 Taiwanese children at the age of 3 years. This is one of the most comprehensive studies in this field, enrolling a large, socio-demographically diverse sample of Mandarin-speaking children within their first 3 years of life. We hypothesized that psychological stress might disrupt the children’s immune system, thus increasing the risk of OM.

## Materials and methods

### Subjects

The present study was performed as a part of the “Kids in Taiwan: National Longitudinal Study of Child Development & Care (KIT) Project” [43], a prospective cohort study for surveying the characteristics of childcare and development. The KIT project included the baseline data of a nationally representative sample collected between 2016 and 2017; there was no significant difference in sex distribution between the study sample (n = 1998) and the population (N = 195601). In this study, we analyzed children who were 3 years of age between 4/1/2016 and 3/31/2017. This cohort is scheduled to be followed up annually until 8 years of age. This study was approved by the research ethics committee of National Taiwan University (No. 201408ES007). Informed consent was obtained from the participating families.

### Data collection

**Fig 1:**
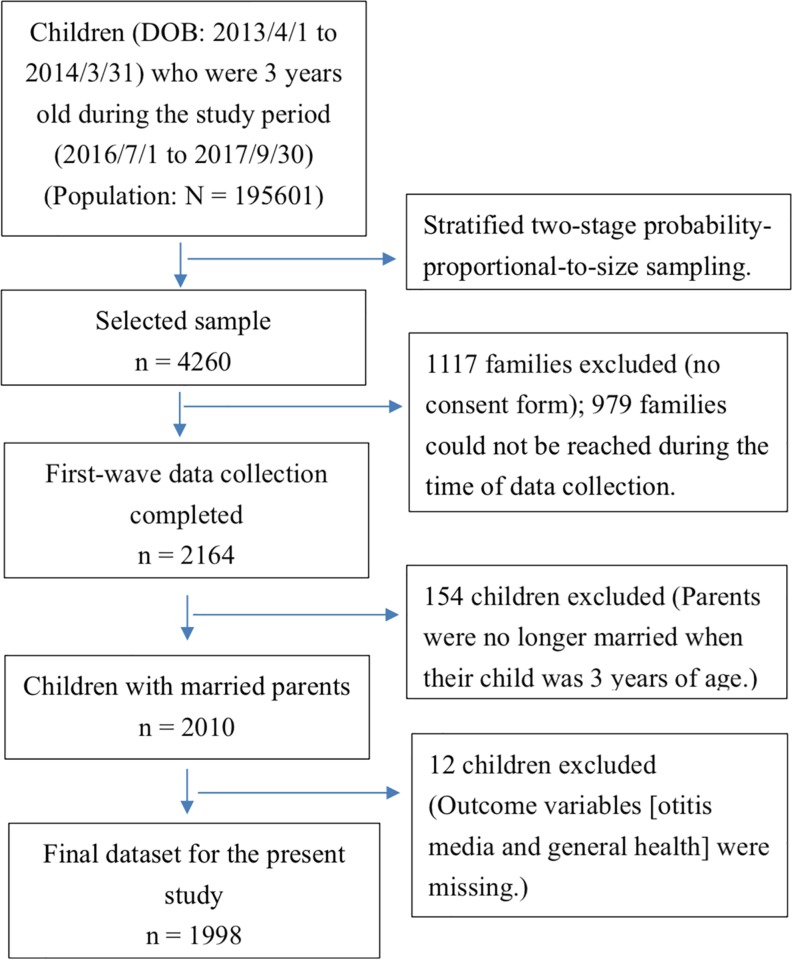
Flowchart of participant selection.

The KIT project used the national census registry as the sampling frame. Potential participants were sampled by using a stratified two-stage probability-proportional-to-size sampling method, with county and person as the primary and secondary sampling units. Once the sample was received, the individuals were sent invitation letters with information about the project. Fig 1 presents the flowchart of participant selection. Of the 4260 individuals in the sample, data of 1998 children were analyzed. Pre-trained personnel with good understanding of the questionnaires [44] visited each family and performed the survey with the parent or guardian who knew the most about the child’s health and healthcare experiences. Each survey was responded by only one person such as the child’s father, mother, or grandparent, hereafter referred to as “parent”. If the respondents had any questions, our pre-trained personnel would provide more detailed information to assist them in understanding and answering the questionnaires [44].

### Definition of outcome variables

#### Parent-reported OM

The primary outcome was parent-reported episodes of OM during the first 3 years of life. Parents were asked the following question about child OM: “has the child ever been diagnosed with OM since birth?” The response options were: “none,” “yes, __ times”, and “I don’t know.” To assess whether OM was present, the outcome was dichotomized as “no OM” or “1 or more episodes of OM.”

#### Parent-reported child health

The secondary outcome for this study was child health status. It was assessed by the question: “in your opinion, what is the current health condition of the child?” Parents could choose among the following responses: (1) the child has a very serious health issue; (2) the child gets sick very often; (3) the child’s health condition is normal; he/she gets sick occasionally; or (4) the child is very healthy. The results were dichotomized as average to poor (responses 1 to 3) versus very healthy (response 4).

### Definition of risk factors

The risk factors of interest in this study, including child characteristics, stress from changing caregivers, parental pressure, parenting style, and child behavior, were assessed through parent questionnaires. Under child characteristics, we examined the occurrence of complications at birth as well as the duration of breastfeeding, which was categorized as follows: (1) the child has never been breastfed; (2) the child was breastfed for less than a month; (3) the child was breastfed for __ months; and (4) the child is still being breastfed.

To assess stress from changing caregivers, the following question was used to calculate the number of times the child changed caregiver: “how many different non-parental caregivers have been taking care of this child?” If the child had never been under the care of a person other than his parent(s), this item was coded “0”. Another question concerned daycare attendance: “is the child receiving care from a center-based setting, such as a nursery care center, a nanny service, or a preschool?” Parents could answer “yes” or “no”.

The indicators of parental pressure examined in this study included parental educational level and mental health, family poverty, employment status of the parents, marriage satisfaction, co-parenting, and family structure. For marriage satisfaction and co-parenting, the survey asked how much parents agreed with the following sentences: “I am satisfied with my marriage,” and “my spouse and I work together as a team in educating our child/children” respectively. The parents scored the sentences from 1 to 4, with 1 meaning “strongly disagree” (the item is 90%~100% inconsistent with the actual condition) and 4 “strongly agree” (the item is 90%~100% consistent with the actual condition). We assessed the mental health of the parents by asking: “In the past 3 months, have you felt sad, depressed, melancholic, or unhappy?” The parents responded by answering “never,” “rarely,” “sometimes,” or “often;” this variable was then dichotomized as “rarely” or “sometimes.” To a question on whether “the income of the family is sufficient for paying living expenses,” the parents answered with a score of 1 to 4, with 1 indicating “strongly disagree” and 4 indicating “strongly agree.”

To address the hypothesis that parenting style is related to higher OM incidence and poor child health, parents were asked to score the following statements to judge the level of harsh discipline: (1) I scold the child if he/she is not obedient; (2) when necessary, I discipline the child by limiting his/her actions; and (3) when the child is out of control, I spank him/her. They answered by scoring each statement on a 4-point scale as follows: 1, rarely (never or less than once a week); 2, sometimes (once or twice a week); 3, often (three to four times a week); or 4, very often (five to seven times a week). The sum of each score was considered as the total harsh discipline score, with higher scores denoting a greater degree of discipline.

Regarding child behavior, several questions adapted from other studies were used to assess negative affectivity, surgency or extroversion [45], inhibitory control [46], and aggressive behaviors [47]. These questions use a 5-point scale. The scores for each item are summed to get the total score for each trait.

### Psychometric validity of outcome measures

Validity analyses were performed using Pearson's correlation and chi-squared tests for test-retest reliability of harsh parenting scores (r = 0.80, p < 0.01), parents’ depressive mood scores (r = 0.61, p < 0.01), and episodes of OM (χ^2^ = 141.08, p < 0.001).

### Statistical analyses

We first examined the relationship between the risk factors identified from previous literature and the outcome variables selected in this study by using the chi-squared test and analysis of variance (ANOVA). Factors that presented a significant relationship with our outcome variables were then analyzed using a hierarchical logistic regression model to examine their explanatory effects on the outcome variables, after controlling for the effects of other factors. The first regression model (Model 1) included parenting style and parental mental health as independent variables. The second regression model (Model 2) included child and family background as additional independent variables. Childcare characteristics and child behavior and temperament were added into Model 3 and 4, respectively. Odds ratios from the logistic regression models and their 95% confidence intervals were used to demonstrate relationships between independent variables and outcomes. All statistical procedures were performed by using SPSS, version 23 (IBM Corp., Armonk, NY, USA).

## Results

### Baseline characteristics of the study population

The baseline characteristics of the study population are reported in Table 1. The proportion of children who had experienced at least one episode of OM during the first 3 years of their life was 12.5%; the remaining 87.5% of the subjects had never had an episode of OM. In terms of general health, 62.7% of the children were very healthy, while 37.3% had average to poor health.

**Table 1:** Selected baseline characteristics of the study sample.

Characteristics	n (%)
Sex	
Male	1015 (50.8)
Female	983 (49.2)
Survey respondents	
Father	345 (17.3)
Mother	1625 (81.3)
Grandparent	28 (1.4)
Child health	
Very healthy	1252 (62.7)
Average to poor	746 (37.3)
Otitis media	
Never	1748 (87.5)
One or more episodes	250 (12.5)
Complications at birth	
No	1419 (71.0)
Yes	579 (29.0)
Duration of breastfeeding	
(Please see S1 Table)	
Maternal education	
Elementary school graduate	17 (0.9)
Junior high school graduate	85 (4.3)
Senior high school graduate	503 (25.3)
College graduate	332 (16.7)
University graduate	826 (41.5)
Master’s degree or higher	226 (11.4)
Sufficient family income	
Strongly disagree	43 (2.2)
Disagree	224 (11.2)
Agree	1323 (66.4)
Strongly agree	402 (20.2)
Caregiver change: number of times	
0	1520 (76.5)
1	199 (10.0)
2	195 (9.8)
≥3	73 (3.8)
Ever attended daycare centers	
No	1434 (72.0)
Yes	558 (28.0)
Co-parenting	
Strongly disagree	38 (1.9)
Disagree	254 (12.9)
Agree	1113 (56.4)
Strongly agree	567 (28.8)
Harsh discipline score	
≤4	117 (5.9)
5–8	1013 (50.9)
9–12	860 (43.1)
Poor maternal mental health	
Sometimes	484 (24.6)
Rarely	1494 (75.4)

### Factors associated with child general health and OM

Table 2 shows the factors associated with our outcome variables. The results of the chi-squared test and ANOVA revealed the following factors to be linked with child general health: complications at birth; daycare attendance; poor paternal and maternal mental health; maternal education level; family financial status; number of caregivers changed; marriage satisfaction; co-parenting; harsh discipline; and child behaviors, including aggression, extroversion, and inhibitory control. In addition, daycare attendance, poor maternal mental health, parental education level, number of caregivers changed, and harsh discipline were related to the occurrence of one or more episode of OM.

**Table 2:** Factors associated with child general health and otitis media.

	General Health		*X*^*2*^ Test	Otitis Media		*X*^*2*^ Test
	Average to Poor (n = 746)	Very Healthy (n = 1252)		One or More Episodes (n = 250)	Never (n = 1748)	
Characteristics	*n* (%)			*n* (%)		
Sex			1.452			0.073
Male	392 (0.53)	623 (0.50)		129 (0.52)	886 (0.51)	
Female	354 (0.48)	629 (0.50)		121 (0.48)	862 (0.49)	
Complications at birth			22.782***			2.027
No	483 (0.65)	936 (0.75)		168 (0.67)	1251 (0.72)	
Yes	263 (0.35)	316 (0.25)		82 (0.33)	497 (0.28)	
Father working status			0.924			0.665
Not full time	30 (0.4)	62 (0.5)		9 (0.04)	83 (0.05)	
Full time	712 (0.96)	1183 (0.95)		240 (0.96)	1655 (0.95)	
Mother working status			0.007			0.205
Not full time	309 (0.42)	524 (0.42)		108 (0.43)	725 (0.42)	
Full time	431 (0.58)	725 (0.58)		142 (0.57)	1014 (0.58)	
Family Structure			2.928			1.257
Two-parent household	689 (0.92)	1175 (0.94)		238 (0.95)	1626 (0.93)	
Not a two-parent household	57 (0.08)	71 (0.06)		12 (0.05)	116 (0.07)	
Ever attended daycare centers			53.917***			13.033***
No	463 (0.62)	971 (0.78)		156 (0.62)	1278 (0.73)	
Yes	279 (0.38)	279 (0.22)		94 (0.38)	464 (0.27)	
Poor paternal mental health			16.323***			0.449
Rarely	167 (0.23)	190 (0.15)		49 (0.20)	308 (0.18)	
Sometimes	569 (0.77)	1041 (0.85)		200 (0.80)	1410 (0.82)	
Poor maternal mental health			26.796***			8.392**
Rarely	231 (0.31)	257 (0.21)		80 (0.32)	408 (0.24)	
Sometimes	512 (0.69)	982 (0.79)		170 (0.68)	1324 (0.76)	
	Mean (SD)	Mean (SD)	F test	Mean (SD)	Mean (SD)	F test
Months of breastfeeding (0–36)	8.77 (9.28)	9.22 (9.45)	1.232	9.44 (9.17)	8.98 (9.42)	0.513
Father’s education (1–6)	4.34(1.21)	4.26 (1.22)	1.744	4.47 (1.19)	4.27 (1.22)	5.980*
Mother’s education (1–6)	4.34 (1.11)	4.24 (1.15)	3.934*	4.48 (0.98)	4.25 (1.16)	8.781**
Family Financial status (1–4)	2.99 (0.63)	3.08 (0.63)	7.962**	3.05 (0.62)	3.05 (0.63)	0.024
Number of caregivers changed (0–10)	0.58 (1.05)	0.33 (0.77)	37.465***	0.60 (1.03)	0.40 (0.87)	10.776**
Marriage satisfaction (1–4)	3.28 (0.58)	3.37 (0.59)	10.485**	3.30 (0.58)	3.34 (0.59)	1.154
Co-parenting (1–4)	3.02 (0.68)	3.18 (0.69)	22.702***	3.11 (0.69)	3.12 (0.70)	0.123
Harsh discipline (3–12)	8.21 (2.17)	7.92 (2.25)	7.707**	8.45 (2.15)	7.96 (2.22)	10.520**
Aggressive behaviors (4–20)	6.43 (2.32)	5.99 (2.15)	19.157***	6.40 (2.26)	6.12 (2.22)	3.398
Surgency or extroversion (3–15)	10.60 (2.31)	11.16 (2.33)	27.026***	10.78 (2.24)	10.98 (2.35)	1.564
Inhibitory control (4–20)	15.05 (3.07)	15.98 (2.83)	47.531***	15.66 (2.86)	15.62 (2.97)	0.040
Negative affectivity (4–20)	13.06 (2.86)	12.90 (3.07)	1.3318	13.27 (2.97)	12.91 (3.00)	3.116

*p < 0.05

**p < 0.01

***p < 0.001. SD: standard deviation.

### Model 1: Relationship of child health and OM with parenting styles and parental mental health

A hierarchical logistic regression analysis was performed to determine the best model describing the relationship of sources of psychological stress with child general health and OM. Co-parenting (odds ratio [OR]: 0.753; 95% confidence interval [CI]: 0.633–0.897) increased the odds of better child health, while harsh discipline (OR: 1.049; 95% CI: 1.005–1.095) and maternal mental health (OR: 1.433; 95% CI: 1.083–1.896) were associated to poorer child general health (Table 3). Harsh discipline (OR: 1.091; 95% CI: 1.025–1.161) and maternal mental health (OR: 1.913; 95% CI: 1.315–2.784) were associated to OM (Table 4).

**Table 3:** Hierarchical logistic regression analysis for the general health of the children.

	Average to Poor vs. Very Healthy			
	Odds Ratio (95% Confidence Interval)			
	Model 1	Model 2	Model 3	Model 4
Marriage satisfaction	1.023 (0.834–1.256)	1.039 (0.841–1.283)	1.040 (0.840–1.289)	1.117 (0.898–1.390)
Co-parenting	0.753** (0.633–0.897)	0.748** (0.627–0.892)	0.755** (0.632–0.903)	0.778** (0.649–0.933)
Harsh discipline	1.049* (1.005–1.095)	1.050* (1.005–1.096)	1.051* (1.006–1.098)	1.057* (1.010–1.106)
Father’s mental health	1.122 (0.823–1.528)	1.126 (0.823–1.540)	1.136 (0.826–1.562)	1.110 (0.803–1.533)
Mother’s mental health	1.433* (1.083–1.896)	1.417* (1.069–1.879)	1.451* (1.089–1.933)	1.404* (1.049–1.881)
Complications at birth		1.592*** (1.296–1.957)	1.577*** (1.279–1.944)	1.530*** (1.236–1.893)
Father’s education		1.042 (0.940–1.156)	1.031 (0.928–1.145)	1.022 (0.918–1.137)
Mother’s education		1.111 (0.995–1.241)	1.066 (0.952–1.193)	1.061 (0.946–1.190)
Family financial status		0.875 (0.740–1.036)	0.878 (0.740–1.041)	0.873 (0.733–1.040)
Changing caregivers			1.163* (1.029–1.314)	1.164* (1.030–1.316)
Daycare attendance			1.846*** (1.453–2.345)	1.880*** (1.475–2.396)
Aggressive behaviors				1.074** (1.027–1.122)
Surgency or extroversion				0.922** (0.880–0.965)
Inhibitory control				0.927*** (0.894–0.961)
-2LL	2458.131	2427.917	2372.583	2315.965
Omnibus test χ^2^	44.865***	30.214***	55.334***	56.617***
Hosmer–Lemeshow test χ^2^	15.501	13.855	7.031	2.044

*p < 0.05

**p < 0.01

***p < 0.001.

**Table 4:** Hierarchical logistic regression analysis for otitis media development.

	Yes vs. No			
	Odds ratio (95% Confidence Interval)			
	Model 1	Model 2	Model 3	Model 4
Marriage satisfaction	0.842 (0.628–1.131)	0.835 (0.616–1.130)	0.832 (0.614–1.129)	0.839 (0.618–1.140)
Co-parenting	1.132 (0.877–1.461)	1.103 (0.854–1.425)	1.116 (0.862–1.444)	1.121 (0.865–1.453)
Harsh discipline	1.091** (1.025–1.161)	1.093** (1.027–1.164)	1.094** (1.027–1.165)	1.091** (1.024–1.163)
Father’s mental health	0.692 (0.449–1.066)	0.715 (0.463–1.105)	0.712 (0.459–1.103)	0.716 (0.462–1.110)
Mother’s mental health	1.913** (1.315–2.784)	1.913** (1.313–2.785)	1.940*** (1.329–2.832)	1.900*** (1.300–2.778)
Complications at birth		1.172 (0.874–1.571)	1.163 (0.867–1.560)	1.165 (0.867–1.564)
Father’s education		1.100 (0.946–1.278)	1.092 (0.938–1.271)	1.088 (0.934–1.267)
Mother’s education		1.134 (0.965–1.334)	1.103 (0.936–1.300)	1.096 (0.930–1.293)
Family financial status		0.954 (0.751–1.213)	0.957 (0.753–1.216)	0.946 (0.743–1.204)
Changing caregivers			1.068 (0.917–1.244)	1.070 (0.918–1.246)
Daycare attendance			1.475* (1.063–2.046)	1.475* (1.063–2.047)
Aggressive behaviors				1.033 (0.971–1.099)
Surgency or extroversion				0.970 (0.910–1.033)
Inhibitory control				1.009 (0.959–1.062)
-2LL	1428.429	1417.203	1407.209	1405.317
Omnibus test χ^2^	21.813**	11.226*	9.994**	1.892
Hosmer–Lemeshow test χ^2^	4.097	14.881	8.881	8.361

*p < 0.05

**p < 0.01

***p < 0.001.

### Model 2: Addition of child and family background

The relationship of child general health with complications at birth (OR: 1.592; CI: 1.296–1.957) was significant (Table 3). On the other hand, neither this factor nor the duration of breastfeeding showed a significant relationship with OM (Table 4; data on breastfeeding not shown).

### Model 3: Addition of childcare characteristics

The number of changes in caregivers and daycare attendance were significantly associated with poor child general health (Table 3). While daycare attendance was also associated with OM (OR: 1.475; 95% CI: 1.063–2.046), the number of changes in caregivers was not (OR: 1.068; 95% CI: 0.917–1.244; Table 4).

### Model 4: Addition of child behavior and temperament

Aggressive behavior (OR: 1.074; 95% CI: 1.027–1.122), extroversion (OR: 0.922; 95% CI: 0.880–0.965), and inhibitory control (OR: 0.927; 95% CI: 0.894–0.961) showed a significant relationship with child general health (Table 3). In contrast, there was no relationship between OM and aggressive behavior, extroversion, inhibitory control, or negative affectivity in the children (Table 4).

## Discussion

The incidence of OM by the age of 3 years in Taiwan was found to be 12.5%. Our results confirmed that daycare attendance and predictors of high psychological stress in children—including harsh discipline and poor maternal health—are related to parentally reported OM in children aged 3 years. It is important to note that most risk factors retained an independent association with health and OM in mutually adjusted models. This suggests that each factor represents a unique source of health risk or vulnerability to OM. On the other hand, we found no association between OM and duration of breastfeeding, insufficient family income, or parental education level.

### Parent-reported OM incidence in Taiwan

Although previous studies have reported under- or over-estimation of OM incidence when using parental reports [48, 49], we believe our study provides a good estimate of OM incidence in Taiwanese children. Our data were collected by professionally-trained personnel who visited each family and conducted the survey. If parents had any question about OM, our trained personnel would provide more detailed information to ensure that the OM cases reported were actual diagnoses made by physicians. Parents, therefore, would have a low probability of confusing OM with other ear infections, and parent-reported OM should correlate well with medical records.

The incidence rate of OM in Taiwan is lower than in other countries. Our result reported a 12.5% rate of experiencing at least one episode of OM during the first 3 years of life. Even though several studies from the US [3–5, 10] found a much higher incidence rate, studies conducted in Taiwan [7, 8] also reported a low incidence of OM. Ting et al. analyzed the incidence of OM from different databases [8]; according to the two questionnaire-based databases, only 7.46% and 9.21% of children before the age of 5 years had experienced at least one episode of OM. The third database, using representative data extracted randomly from the entire National Health Insurance Research Database (NHIRD) in Taiwan, reported that only 3% of infants had had OM by the age of one year. This result was very different from that of a survey conducted in the US, where 25%– 36% of children have experienced at least 1 episode of acute OM by the age of one year [3]. One explanation for the low incidence of OM in Taiwan is the high rate of antibiotics overuse [50, 51]. According to Chang et al., Taiwanese pediatric patients often receive antibiotic treatment for viral infections, and more than 30% of patients diagnosed with the common cold receive antibiotic treatment [51]. This trend of antibiotics overuse could act as prophylaxis for the prevention of OM development, or as a treatment for early OM before physicians could make the diagnosis.

### Relationship between breastfeeding and OM

The preventive effect of breastfeeding on OM has been widely studied. Several studies [13, 52] have shown that even a short duration of breastfeeding might be beneficial for preventing OM. However, in our analysis, the protective effect of breastfeeding against OM was not evident. This discrepancy might be due to the fact that we did not assess feeding behaviors such as the proportion of breast milk and the use of pumped milk. In a study conducted by Boone et al., only a few mothers had fed their children the same substance by the same mode [53]; most mothers used 100% breast milk at one time and a mixture of formula and breast milk at another. Since the benefit of breast milk is dose-dependent, non-exclusive breastfeeding would provide less protection for children [54]. Furthermore, in Taiwanese culture, mothers tend to feed their children pumped milk through baby bottles. Pumped milk has greater contamination [55] and lower nutritional value (energy and fat concentration) [56] and immunologic activity [57] than breast milk. Therefore, the children included in our study might have received less protection against OM because of non-exclusive breastfeeding and the use of pumped milk.

### Relationship between daycare attendance and OM

Several studies have already reported an association between out-of-home care and an increased risk of OM [5, 14, 58, 59]. The most commonly accepted explanation for this association is that frequent close interpersonal contacts result in a greater microbial load exposure and thus a greater risk of OM [17, 60–62]. However, since the pathogenesis of OM is multifactorial, involving not only the microbial load in the environment but also the host’s immune response [5], we suspect that the psychological stress imposed by daycare attendance might be one of the factors that causes OM by impairing the host’s immune response.

Children may experience stress when attending daycare centers. When transitioning from homecare to daycare centers, children face several challenges such as adjusting to new daily routines, separating from familiar caregivers, and learning new social interactions. This stress is reflected in their increased cortisol levels [29]. Bernard et al. found that children experience an increase in cortisol levels when they are transitioned from homecare to out-of-home childcare [32]; in fact, the effect of daycare attendance on cortisol level was especially significant in children younger than 3 years [30]. In addition, several other immune responses are disrupted when children are under psychological stress. The levels of interleukin 1, interleukin 8, and tumor necrosis factor (TNF)—which are part of the immune response to OM [5]—are disrupted when children are under stress [20]. Although it has not been shown that an increase in cortisol levels or an imbalance in cytokine levels are predictive of OM, it is still reasonable to suspect that the disruption of neuroendocrine function and immunity might make children more vulnerable to OM. This may be one of the reasons why some children get OM, while others do not, when all children are exposed to a similar microbial load in a daycare center; however, further research is needed to investigate this relationship.

### Relationship of parental mental health and parenting style with OM

Consistent with previous findings, our results showed that poor maternal mental health and harsh discipline cause stress in children and have a negative impact on their health [25, 41, 63, 64]. Our results further suggest that both these factors are related to OM. Paternal mental health, however, was not associated with a higher incidence of OM. There are two explanations regarding why poor maternal, but not paternal, mental health is predictive of a greater risk of OM. First, in our study, fathers reported feeling “depressed sometimes” (18.1%) less frequently than did the mothers (75.4%). Additionally, mothers are predominantly the primary caregivers in traditional Taiwanese households. A fewer number of depressed fathers and their lower contribution to childcare might, therefore, have led to a non-significant relationship between paternal mental health and OM.

As the main caregivers for children, mothers with poor mental health could cause psychological stress to their children in several ways. Children born to parents with depressive symptoms might be genetically more vulnerable to depression as well, and a previous study has shown that daughters with a familial risk for depression tend to have a greater cortisol reactivity to stress [24]. Therefore, according to our hypothesis, these mentally stressed children might have disrupted immunity and a higher incidence of OM. Another possibility is that depressed mothers might exhibit poor childcare quality and mother–child interactions, or neglectful or harsh parenting [65]; neglected or maltreated children are known to be at risk for physical disorders in childhood [66]. Furthermore, maternal psychological stress and harsh discipline have also been found to be associated with increased cortisol levels in children [27]. In short, children can have a higher risk of OM because of inherited depressive genes or poor parenting from depressed mothers.

### Differences in risk factors for OM and child health

Our results show that an increase in the number of caregiver changes and less co-parenting predispose to poor child health, but not to OM. A possible explanation is that the parental subjective view of the child health may be worse in parents who cooperate less in child care. Change of caregivers and co-parenting may be indicators of cooperation between the parents and of the quality of child care; parents may think that if there is more cooperation in child care, the child general health could be in better status. A similar idea has been discussed in other studies, showing that single-parent or -grandparent families may show lower quality of child care and thus poorer child health [67, 68]. The child general health considered in this study is a subjective measure held by the parents, so children’s general health is viewed negatively by the less-cooperate parents. OM, on the other hand, is a relatively more objective measure of a child’s disease; our trained personnel can ensure that the parent-reported cases of OM were actually diagnosed by a physician. It is thus a valid measure of the child’s actual health condition that is not influenced by the subjective point of view of the parents. In short, we hypothesize that change of caregivers and co-parenting influence general health but not OM because the former is a subjective measure while the latter is objectively assessed.

Child behavior and temperament were found to be related to child health but not to OM. Several factors affect child temperament, including age, gender, social and cultural factors, and parental characteristics [69]; anxious or depressed mothers tend to report more child behavior problems [25, 70]. Consequently, mothers who rate their child as having poor general health could be anxious, and thus report more child behavior or temperament problems. In contrast, OM is an acute disease episode, typically occurring only few times, which could have a smaller impact on the long-term anxiety of mothers, and thus on the reports of child behavior and temperament.

### Strengths and limitations

The current study has several strengths. Because our nationally representative study sample was large in size, and the KIT database provided detailed information on the social and environmental contexts in which Taiwanese children live, we believe our findings can be extrapolated to most Mandarin-speaking societies. More importantly, this study adds to the existing literature by showing that psychological stress in children could potentially be a predictive factor for the risk of OM; however, further research is needed to confirm the causal relationship.

Our study also has some limitations. First, parent-reported OM may not be 100% accurate. However, several studies have shown parent reports to be an acceptable source of information for children health and disease [71–74]; in particular, D’Souza-Vazirani et. al found parental reports to be an accurate source of information for child acute health care events in the first 3 years of life [71]. Although the accuracy of parent-reported OM history over 1 to 2 years was only fair [73], we did not need parents to recall all the OM details. Our study only asked whether the child ever had OM or not before the age of 3 years. The severity or frequency of OM was not investigated, and this could minimize parent recall problems in our estimate of OM incidence. Second, certain potentially important risk factors were not investigated in this study; some examples include the use of a baby bottle and the daycare center group size. Third, this study only investigated association and not causation. This limitation will be addressed to some extent in our next project, which involves both parents and children (at the age of 4 years) remaining in the study. By repeating our analyses on a longitudinal dataset, we might gain a better understanding of the links between OM and childhood stress. The fourth limitation stems from the fact that not many studies have reviewed the underlying mechanism of OM infection in psychologically stressed children. More research is needed to investigate the effect of hormonal and immunological changes in children under high psychological stress on the development of OM.

## Conclusions

This study is the first to document a significant relationship between childhood OM and sources of psychological stress, including daycare attendance, poor maternal mental health, and harsh discipline. Our findings provide a new perspective on the possible risk factors for OM development. Adequate psychosocial support to both parents and children might be a new strategy for preventing OM. However, further studies are necessary to address the hormonal and immunological mechanisms of psychological stress in OM development.

## Supporting information

S1 Table Months of breastfeeding. (DOCX)
