# Supplementary material for: Childhood otitis media: Relationship with daycare attendance, harsh parenting, and maternal mental health
Source: PLoS One. 2019 Jul 16;14(7):e0219684. doi: 10.1371/journal.pone.0219684 (PMC6634415; doi:10.1371/journal.pone.0219684)
Supplement: S1 Table — (DOCX) [file pone.0219684.s001.docx]

| Months of Breastfeeding | | | | | |
| --- | --- | --- | --- | --- | --- |
|  | | Number | Percentage | Effective percentage | Cumulative Percentage |
|  | .00 | 130 | 6.5 | 6.5 | 6.5 |
|  | .50 | 251 | 12.6 | 12.6 | 19.2 |
|  | 1.00 | 106 | 5.3 | 5.3 | 24.5 |
|  | 1.50 | 12 | .6 | .6 | 25.1 |
|  | 1.90 | 1 | .1 | .1 | 25.1 |
|  | 2.00 | 138 | 6.9 | 6.9 | 32.1 |
|  | 2.50 | 3 | .2 | .2 | 32.2 |
|  | 3.00 | 99 | 5.0 | 5.0 | 37.2 |
|  | 4.00 | 76 | 3.8 | 3.8 | 41.0 |
|  | 4.50 | 1 | .1 | .1 | 41.1 |
|  | 5.00 | 33 | 1.7 | 1.7 | 42.7 |
|  | 6.00 | 218 | 10.9 | 11.0 | 53.7 |
|  | 6.50 | 1 | .1 | .1 | 53.7 |
|  | 7.00 | 56 | 2.8 | 2.8 | 56.6 |
|  | 7.50 | 1 | .1 | .1 | 56.6 |
|  | 8.00 | 87 | 4.4 | 4.4 | 61.0 |
|  | 9.00 | 36 | 1.8 | 1.8 | 62.8 |
|  | 9.50 | 1 | .1 | .1 | 62.8 |
|  | 10.00 | 56 | 2.8 | 2.8 | 65.7 |
|  | 11.00 | 27 | 1.4 | 1.4 | 67.0 |
|  | 11.50 | 1 | .1 | .1 | 67.1 |
|  | 12.00 | 191 | 9.6 | 9.6 | 76.7 |
|  | 13.00 | 21 | 1.1 | 1.1 | 77.7 |
|  | 14.00 | 38 | 1.9 | 1.9 | 79.6 |
|  | 15.00 | 51 | 2.6 | 2.6 | 82.2 |
|  | 16.00 | 14 | .7 | .7 | 82.9 |
|  | 17.00 | 7 | .4 | .4 | 83.3 |
|  | 18.00 | 63 | 3.2 | 3.2 | 86.4 |
|  | 19.00 | 8 | .4 | .4 | 86.8 |
|  | 20.00 | 9 | .5 | .5 | 87.3 |
|  | 21.00 | 5 | .3 | .3 | 87.5 |
|  | 22.00 | 11 | .6 | .6 | 88.1 |
|  | 23.00 | 3 | .2 | .2 | 88.2 |
|  | 24.00 | 78 | 3.9 | 3.9 | 92.2 |
|  | 25.00 | 2 | .1 | .1 | 92.3 |
|  | 26.00 | 7 | .4 | .4 | 92.6 |
|  | 27.00 | 14 | .7 | .7 | 93.3 |
|  | 28.00 | 9 | .5 | .5 | 93.8 |
|  | 29.00 | 5 | .3 | .3 | 94.0 |
|  | 30.00 | 22 | 1.1 | 1.1 | 95.1 |
|  | 31.00 | 4 | .2 | .2 | 95.3 |
|  | 32.00 | 4 | .2 | .2 | 95.5 |
|  | 33.00 | 2 | .1 | .1 | 95.6 |
|  | 34.00 | 6 | .3 | .3 | 95.9 |
|  | 35.00 | 1 | .1 | .1 | 96.0 |
|  | 36.00 | 80 | 4.0 | 4.0 | 100.0 |
|  | Total | 1989 | 99.5 | 100.0 |  |
| Loss |  | 9 | .5 |  |  |
| Total | | 1998 | 100.0 |  |  |
